# September

**Published:** 1890-09-06

**Authors:** 


### SEPTEMBER.

On the 1st of September, as everyone knows, partridge shooting commences. Well, this is a practice which we suppose, in England at least, will never die out. But we are old-fashioned enough to wish that it were carried on as our grandfathers pursued it. They gave the birds a chance, although they prided themselves on their skill with the gun. They did not have birds driven up to them, they walked after the birds. And they enjoyed a long day on the stubble or through the turnips, marking down their birds, and obtaining real sport, if only a single covey were put up on a farm. Neither was it usual for them to be accompanied by ladies. Now, however, it is different, and oftentimes ladies follow "the rude clamour of the sportsman's gun," and have even a special costume for the sport, on this occasion *not* taking copy from the goddess Diana, whose clothes were scanty and not exactly suited for British thorns and thickets. No doubt exercise is a very good thing for women as well as for men. It is on record that a certain African potentate so protected his female belongings that they got no exercise, and had nothing to do, the consequences being that the ladies grew obese, diseased, and then died. So the potentate had to replenish his stores about every third year. We do not desire that English ladies should pass their time as of yore, in producing hideous images of impossible birds or flowers in wool work; but we think there is much more suitable occupation and amusement for them than rambling with men over stubbles and turnips after partridges. Although we do not think that Omphale always looks best with a distaff, we certainly do think that Hercules looks best with the club!

Brown autumn commences now to shed her sober tints upon the woods. But the beauty of the country is scarcely more attractive than when decaying, herein differing from the beauty of the female form divine. The long grass is now dark in shady places, for there "early falls the dew at eve," and lingers long after sunrise. Already the first leaves—the early spoil of the season—lie upon the ground. Summer is fading fast, although the corn-marigold and the crimson poppy yet linger on the yellow stubble. But autumn is "laden with the richest products of the earth," the garners are full, and the people may rejoice!

September was the seventh month of the old Roman year. By the Julian arrangement, while retaining its name and number of days it became the ninth month. In September the mornings decrease by forty-seven minutes, and the evenings by one hour and seven minutes. September 1st is St. Giles' Day. St. Giles was a charitable personage who even sold his coat to enable him to give to the poor. He was esteemed the special patron of cripples, as he would not be cured of a lameness. Hence the old saying, "as lame as St. Giles at Cripplegate." Even before the Conquest cripples used to assemble to ask alms at Cripplegate. Many hospitals, especially for lepers, were dedicated to St. Giles. September 2nd is the anniversary of the Great Fire of London in 1666. Then 89 churches, the City gates, the Guildhall, hospitals, schools, libraries, and 13,200 dwelling-houses were destroyed. The fire began in a baker's house in Pudding Lane, and extended for four days over 436 acres. Some erroneously attributed it to Papist malignancy. Probably we may be now thankful that the fire did occur, for old London would have been still more unsanitary than the modern city. September 3rd is the anniversary of the once famous "Bartlemy Fair," which was discontinued in 1826. Here all kind of "shows" were to be seen, stout ladies, dwarfs, Malays, peep-shows, tight-rope dancers, &c., &c., and both Wombwell's and Richardson's establishments, which long survived Bartlemy Fair. An old chronicler of the Fair mentions that a quaint looking individual added the proverbial skeleton to the joviality by distributing bills headed, "Are you prepared to die?" just as enthusiasts now take advantage of similar merry-makings for the "word in season," or rather, it should be said, out of season. The 3rd of September is also noticeable as the first of the days omitted from the Calendar of the year 1751. Then the time was altered according to the Gregorian style, and the days between the 2nd and 14th of September were omitted, the 3rd becoming the 14th. September 7th is the day of St. Enurchus, who extinguished a fire by the efficacy of his prayers, which threatened to consume Rome. Now we employ the fire brigade! Enurchus was sent from Rome to Orleans in order to counsel the people as to their choice of a bishop. While thus employed a dove descended on his head, "affording to the wondering multitude incontestible proof that he was the proper person." Detractors have asserted that the dove had been trained to the performance! September 8th is the nativity of the Virgin Mary. For some centuries the Church took little notice of the Virgin Mary. But a devout person, hearing a concert of angels, prayed for information, and was told it was the angels celebrating the nativity of the Virgin. Therefore Pope Servius, in 695, appointed a yearly feast. Many are the ancient legends regarding the Virgin. At one time she descended from heaven for the purpose of mending the gown of Thomas A'Beckett. She is also said to have fed St. Allen when indisposed in reward for his devotional attentions, by graciously affording that nourishment which females are accustomed only to give their offspring. On this John Brady, in his "Analysis of the Calendar" (1815) truly remarked, "To what depths of impious absurdity will not ignorance and credulity debase mankind!" About the 10th or 11th of September lunar rainbows may often be seen. September 14th was known as Holyrood-day or Holy-Cross day, or the day of the exaltation of the cross. Rood and cross were synonymous in the old Saxon. It is supposed to be the anniversary of the recovery of the piece of the cross by the Emperor Heraclius, which had been taken away on the plundering of Jerusalem by Chosroes, King of Persia, A.D. 615. Afterwards the Bishop of Jerusalem exhibited it annually to all devout pilgrims. Formerly it was the custom to go a-nutting on this day. Poor Robin, in 1709 said—

> "The devil as the common people say,
> Doth go a-nutting on Holyrood day!"

Perhaps this was with reference to various mischiefs which young people committed when out "a-nutting."

On September 20th, '54, was fought the battle of Alma. At half-past one the British infantry advanced into action. The village of Burliesk, the centre of the British position, was in flames. To the right of the conflagration the Welsh 41st, and the Hertfordshire 49th crossed the river's deep and dangerous ford, and under a galling fire from the Russians. On the extreme left the light division under Sir George Brown crossed the stream and scaled the rugged precipitous banks. Dense vineyards and abattis of felled trees obstructed the advance, and Minie balls showered past like hail. A deadly sheet of fire tore through the 7th Fusiliers—they wavered, but reformed. On rolled the human surge, the 19th, the 33rd, the 77th, the 88th, with colours flying and loud hurrahs. Their leader falls, and for a moment the advance is paralysed. But the grand old soldier is soon at their head on foot. Man grapples with man. Sturdy British with stout Russians. Nine hundred officers and men of England's army fall, but the Red Dragon of the Royal Welsh floats over the great redoubt. Then from the higher hills a mighty column of Russians descend, bearing with them the image of St. Sergius. The ranks of scarlet waver, and some-one exclaimed, "The Guards will be destroyed!" But Colin Campbell said, "Better so, than that Her Majesty's guards should turn their backs to the enemy!" The ground they had to ascend was steep and broken. But the Black Watch, the Cameron and Sutherland Highlanders, surmount the difficulty. A murderous fire is opened upon them, but they still advance, the Russian infantry break and fly, and the heights of Alma are won.

September 21st is St. Matthew's day—the Matthew, otherwise called Levi, who wrote the Gospel in Judæa, some short time after the ascension of Him, whose transcendant merits and endurances form the fundamental basis on which the Christians form their hopes of redemption.

September 29th, is Michaelmas day, sacred to St. Michael and all the angels. It is also quarter day, which many think comes round too quickly. Churchill said: "September, when by custom right divine, geese are ordained to bleed at Michael's shrine." The old custom of goose for dinner on Michaelmas day has not quite died out in country places. It has been stated that the custom had origin from the last word of an old Church prayer *intentos*. The vulgar mistook it to mean a goose with *ten toes*! But it is more probable that as Michaelmas day was a festival day, and the time when geese are plenty, people took to eating them at this season. In the poesies of Gascoigne, 1575, it is stated: "And when the tenants come to paie their quarter's rent, they bring at Michaelmas a goose." Under the headings of several of the months we have referred to the alteration of the Calendar. It is worthy of note that one of the objections made to this alteration was the confusion which would follow if Michaelmas day was not to be celebrated when stubble geese are in in their highest perfection. For,

> "Geese now in their prime season are,
> Which if well roasted are good fare,
> Yet, however friends, take heed,
> How too much on them you feed,
> Lest, when, as your tongues run loose,
> Your discourse do smell of goose."

We think, however, it was the brandy usually taken after goose which turned the discourse. This leads to recollection of the anecdote of the man who being taxed with drinking too much brandy, replied, that he "only tasted it on two occasions, when he had goose for dinner and when he didn't!" An old writer said a goose is "the emblem of modestie." But the modern goose is rather an impudent bird, and does not hesitate to follow and hiss an intruder off his pasture. Geese, indeed, have been known to attack and injure children. Geese, however, were once noted for greater prudence, that is, if we are to believe the old story, that from a consciousness of their own loquacity they always flew over Mount Taurus with stones in their bills to prevent any unlucky cackle betraying them to the eagles.

